# The Community Structure and Diversity of Heterotrophic Microorganisms in the Soils of Taiga Forests, China

**DOI:** 10.3390/microorganisms13081853

**Published:** 2025-08-08

**Authors:** Siyuan Liu, Zhichao Cheng, Mingliang Gao, Libin Yang, Yongzhi Liu

**Affiliations:** 1 Key Laboratory of Biodiversity, Institute of Natural Resources and Ecology, Heilongjiang Academy of Sciences, Harbin 150040, China; liuliu9826@163.com (S.L.); chengzc928@163.com (Z.C.); 2 Heilongjiang Huzhong National Nature Reserve, Huzhong 165038, China; zrbhjzhk@163.com

**Keywords:** taiga forests, soil heterotrophic, microorganisms community structure, biomass, community assembly

## Abstract

Heterotrophic microorganisms derive energy by decomposing organic matter. Their composition and community structure are influenced by environmental factors and interactions. Soil heterotrophic respiration was assessed by establishing vegetation removal plots (Hr) and control plots (Sr). Soil physicochemical properties were analyzed, and the composition and biomass were evaluated using Illumina HiSeq sequencing and PLFA. The pH of Hr exhibited a significant increase (*p* < 0.05), whereas MC, MBC, SOC, DOC, TN, and AN all showed significant decreases (*p* < 0.05). PLFA analysis revealed that the biomass of bacteria, fungi, and total microorganisms in Hr was significantly lower than in Sr (*p* < 0.05). The predominant bacterial phyla were Acidobacteria, Verrucomycota, and Proteobacteria, with Verrucomycota significantly more abundant in Hr. The dominant fungal phyla were Ascomycota and Basidiomycota, both significantly more abundant in Hr. Community assembly was governed primarily by homogeneous selection in both Hr and Sr. The Hr co-occurrence network showed higher complexity, with >60% positive associations. Mantel tests confirmed significant links between soil properties (MC, pH, MBC, SOC, DOC, TN, and AN) and microbial composition. Vegetation removal induced soil heterogeneity and reduced microbial biomass with specific taxa shifts (Verrucomicrobia, Ascomycota, and Basidiomycota). Altered soil conditions and carbon resources reorganize microbial structure and function.

## 1. Introduction

The Greater Xing’an Range Forest Area is situated in the northernmost region of China. It represents the largest contiguous tract of virgin forest in the country and is recognized as the only cold-temperate coniferous forest zone in China. This area constitutes the southernmost extension of the Boreal Forest ecosystem [1]. Forest soil carbon storage constitutes approximately 39% of global soil carbon reserves. As the largest terrestrial carbon reservoir in the Northern Hemisphere, cold-temperate forest soil plays a significant role in the global forest soil carbon pool [2]. In the carbon cycle, soil respiration represents a key flux, and its underlying mechanisms demonstrate distinct characteristics in the cold temperate zone. Environments with lower average annual temperatures tend to suppress the decomposition rate of organic matter [3]. Climate warming may accelerate heterotrophic respiration by enhancing microbial activity, thereby increasing carbon emissions. Specific environmental conditions, such as freeze–thaw cycles, exert significant regulatory effects on microbial activity, leading to distinct soil respiration mechanisms in temperate cold zones compared to those in other climatic regions [4].

Existing studies have demonstrated that soil CO_2_ emissions are primarily derived from two biological processes: heterotrophic respiration (R_h_) and autotrophic respiration (R_a_) [5]. Current research has confirmed that soil respiration intensity is synergistically influenced by various ecological factors, such as climatic conditions, vegetation characteristics, surface cover, and soil physical and chemical properties [6,7]. Bacteria and fungi, being the dominant components of soil microbial communities (accounting for over 90%), play a central role in driving soil respiration [8]. Alterations in soil physicochemical properties indirectly influence respiratory flux by modulating microbial community structure and the bioavailability of organic carbon [9]. Aneesh et al. demonstrated microbial process integration in SOC models, revealing critical microbially mediated decomposition through mechanistic complexity analysis [10]; He et al. established biotic–abiotic linkages to generate a global soil Rh dataset via random forest algorithms for reliable terrestrial carbon sink predictions [11]; and Tao et al. [12]. combined global carbon cycling datasets with microbial process models to elucidate environmentally dependent microbial drivers of carbon cycling. However, existing research primarily focuses on specific ecosystems, such as artificial soil microcosms [13], tropical forest soil [14], agricultural soil [15], and temperate grassland [16]. In contrast, research on the underlying driving mechanisms of Sr and Hr remains limited in the unique environment of cold-temperate forest soils, particularly regarding the synergistic regulation among soil physicochemical properties and microbial communities, which has not yet been systematically investigated.

This study conducted field experiments in the Huzhong National Nature Reserve. By analyzing differences in soil physical and chemical properties (MC, pH, MBC, SOC, DOC, TN, and AN), microbial biomass, and community structure at sites exhibiting Sr and Hr in cold-temperate forests, this research aims to investigate the soil environmental drivers underlying variations in soil respiration and the functional differentiation of key microbial groups. This study addresses a knowledge gap regarding the mechanistic role of soil heterotrophic microorganisms in driving respiration processes within cold-temperate forest ecosystems. Furthermore, it provides a microbiological foundation for optimizing ecosystem carbon turnover models and suggests new directions for carbon sequestration management strategies in cold-temperate forests through microbial functional regulation.

## 2. Materials and Methods

### 2.1. Overview of the Research Site

**Figure 1:**
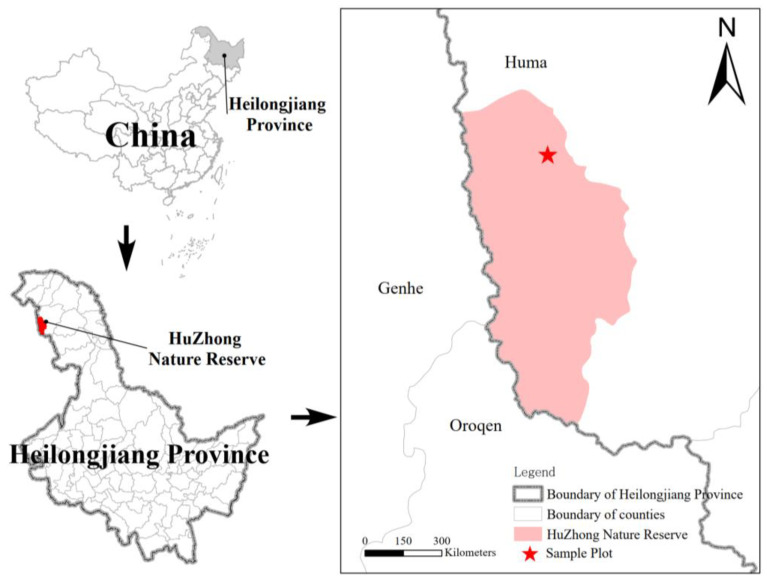
Study area and plot location.

The Huzhong National Nature Reserve (Figure 1) is situated at the northern foothills of the Yilehuli Mountains within the Greater Xing’an Range, spanning longitudes 122°39′ to 124°21′ E and latitudes 51°14′ to 52°25′ N, and covering a total area of 167,213 hm^2^. The topography generally slopes from northwest to southeast, characterized primarily by medium and low altitude mountainous regions with elevations ranging from 400 to 1528 m above sea level. The region experiences a cold temperate continental monsoon climate, marked by long, frigid winters and brief, cool summers. The annual average temperature is approximately −4.3 °C, with annual precipitation varying between 450 and 550 mm, most of which occurs during the summer months. The reserve features a diverse range of soil types, including brown coniferous forest soil, dark brown soil, and meadow soil. Vegetation is predominantly composed of cold-temperate coniferous forests, with major tree species forming dense stands including Larix gmelinii and Pinus sylvestris.

### 2.2. Experimental Plot Establishment and Sample Collection

During the sampling process, a paired sampling strategy was employed to minimize the influence of soil heterogeneity. Initially, at each selected sampling point, an undisturbed soil column (0–20 cm depth) was collected vertically using a stainless steel soil corer (Weichen Technology, Shanghai, China) with an inner diameter of 5 cm. Concurrently, plant root samples with a diameter of ≤2 mm were excavated from the same location and used for measuring total soil respiration (Sr). Additionally, to enhance experimental reliability, a dual control system was implemented. Specifically, a root exclusion zone (established by mechanical root cutting at a depth of 50 cm (the 50 cm buffer depth excludes live fine roots with significant contributions, ensuring result reliability), allows for natural decomposition over a period of six months.) was set up within the sampling plot to serve as a source of heterotrophic respiration (Hr) samples. Soil samples from the root exclusion zone were collected simultaneously with those from the rooted zones. Each treatment group included seven biological replicates, and the sampling points were distributed following a modified serpentine layout method (to avoid systematic bias in fixed sites).

### 2.3. Determination of Soil Physical and Chemical Properties

Soil moisture content (MC) was determined using the oven (Shanzhi Instrument, Shanghai, China) -drying method [17], soil pH was measured via the potentiometric method [18], and soil microbial biomass carbon (MBC) was assessed using the carbon-nitrogen analyzer (AnalytikJena, Thuringia, Germany) method [19]. Soil organic carbon (SOC) and total nitrogen (TN) contents were analyzed with the ELIII (Elementar Vario, Frankfurt, Germany) automatic carbon-nitrogen analyzer [20]. The alkaline diffusion method [21] was employed to determine the contents of dissolved organic carbon (DOC) and soil alkali-hydrolysable nitrogen (AN).

### 2.4. Determination of PLFA Content

The PLFA were detected using the potassium hydroxide–methanol methylation method [22]. A nineteen-carbon alkanoic acid (19:0) was employed as the internal standard during analysis performed on an Agilent 6850 gas chromatograph (Agilent, Wilmington, DE, USA). The phospholipid fatty acid components in the PLFA profiles of soil samples were identified and quantified using the Sherlock MIS 4.0 system (MIDI, Newark, NJ, USA). The concentration of each fatty acid was calculated relative to the 19:0 internal standard and expressed in units of nmol·g^−1^. Specific fatty acid biomarkers indicative of bacterial and fungal biomass in soil were selected based on the references by Jain et al. [23,24,25], and the total PLFA content in the samples was determined according to the following formula:

C_PLFA_ = (A_sample_ × C_IS_ × V_total_)/(A_IS_ × m)

C_PLFA_: PLFA content of the sample (nmol/g); A_sample_: peak area of the target fatty acid; C_IS_: internal standard concentration (nmol/μL); V_total_: total volume of the sample solution (μL); A_IS_: peak area of the 19:0 internal standard; and m: sample mass (g).

### 2.5. Soil Microbial Determination and Analysis

#### 2.5.1. Microbial DNA Extraction

Soil DNA was extracted using the PowerSoil^®^ kit (Sangon Biotech, Shanghai, China), dissolved in 50 μL of deionized water, and stored at −80 °C for subsequent analysis. The bacterial 16S rRNA gene and fungal ITS region were amplified separately using the primer pairs 338F/806R and ITS1/ITS2, respectively. The resulting PCR products were verified via electrophoresis and then combined according to their band intensities (2–8 μL per sample). Following gel extraction and purification, the amplicons were sequenced on the Illumina HiSeq PE2500 (Illumina, San Diego, CA, USA) platform.

#### 2.5.2. Microbial DNA Sequencing and Analysis

After quality control and sorting, the sequences obtained from high-throughput sequencing were processed and analyzed in accordance with the standard operating procedures of Mothur software (v1.39.5). (1) Sequence alignment was conducted using MEGA 7.0 software, following the methodology for constructing a bacterial 16S rRNA gene reference database. (2) The sequence length of each sample was standardized, and sequences that were too short were removed; sequence names were simplified, and barcode sequences were removed. (3) Redundant and chimeric sequences were identified and excluded. (4) Using the constructed bacterial 16S rRNA gene reference database, the taxonomic and species information of the sample sequences was determined. Specifically, each quality-controlled sequence was compared against the 16S rRNA gene reference database of anaerobic ammonia-oxidizing bacteria to identify the most similar reference sequence with a confidence level exceeding 80%. (5) Pairwise sequence similarities were calculated to generate a distance matrix file. (6) Based on this distance matrix, sequences were clustered into ASVs using a similarity threshold of 97%. (7) Low-abundance ASVs were filtered out by removing those represented by fewer than five sequences, and representative sequences of the dominant ASVs were extracted. (8) To eliminate differences in sequencing depth, all samples were homogenized and diluted using QIIME2 (2024.2). The α diversity index was calculated based on the abundance of non-rare ASVs within each sample, while β diversity analysis was performed using Principal Coordinates Analysis (PCoA).

### 2.6. Data Analysis

Statistical analysis of the data was performed using SPSS 21 (Chicago, IL, USA). Significance testing was carried out employing *t*-tests and one-way ANOVA. A *p*-value ≤ 0.05 was considered statistically significant. The results were presented as mean ± standard error (SE) based on seven independent biological replicates. A random forest model was developed using the random forest package in R (version 4.4.1) to identify key microbial taxa. Principal Coordinates Analysis (PCoA), based on Bray–Curtis dissimilarity, and Mantel tests were conducted using the vegan package. βNTI (Beta Nearest Taxon Index) was calculated with the picante package to assess community assembly processes. Collinearity networks were constructed using the linkET and igraph packages. All statistical visualizations were generated using ggplot2, thereby establishing a comprehensive analytical framework for investigating community–environment interactions.

## 3. Results

### 3.1. Physical and Chemical Properties of Soil

The physical and chemical properties of the soil are presented in Table 1. Compared with Hr, the pH of Sr was significantly lower (*p* < 0.05), whereas MC, pH, MBC, SOC, DOC, TN, and AN were all markedly increased (*p* < 0.05). These findings indicate that the removal of vegetation has led to changes in the soil physicochemical properties in cold-temperate forests, demonstrating a certain degree of heterogeneity.

### 3.2. Soil Microbial PLFA Content

**Figure 2:**
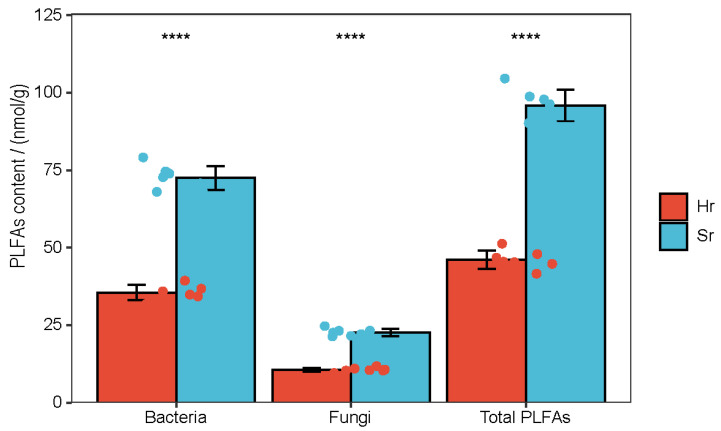
Soil PLFA content varies according to respiratory patterns, (**** *p* < 0.0001).

The PLFA analysis results indicated that, compared with the Hr treatment, the bacterial biomass, fungal biomass, and total microbial biomass in Sr exhibited a highly significant increase (Figure 2).

### 3.3. Microbial Community Composition and Diversity

**Figure 3:**
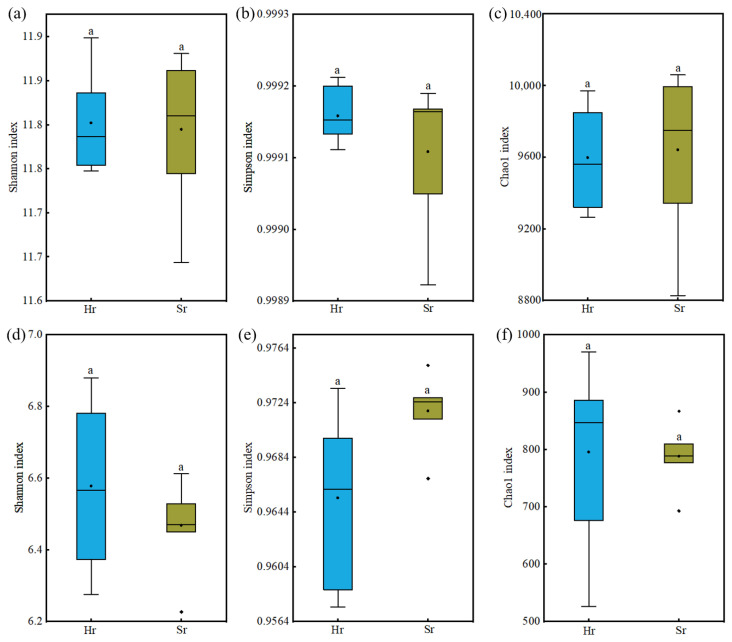
Alpha-diversity of soil microbial communities across different respiration patterns: Shannon index for bacteria (**a**), fungi (**d**), Simpson index for bacteria (**b**), fungi (**e**), Chao1 index for bacteria (**c**), fungi (**f**). Same letters indicate no significant difference.

As presented in Figure 3, no statistically significant differences were observed in the Shannon index, Simpson index, or Chao1 index of α-diversity among microbial communities involved in the two respiration patterns (*p* > 0.05). These findings indicate that two respiration patterns exert minimal influence on microbial alpha diversity; however, inherent differences exist among different microbial groups.

**Figure 4:**
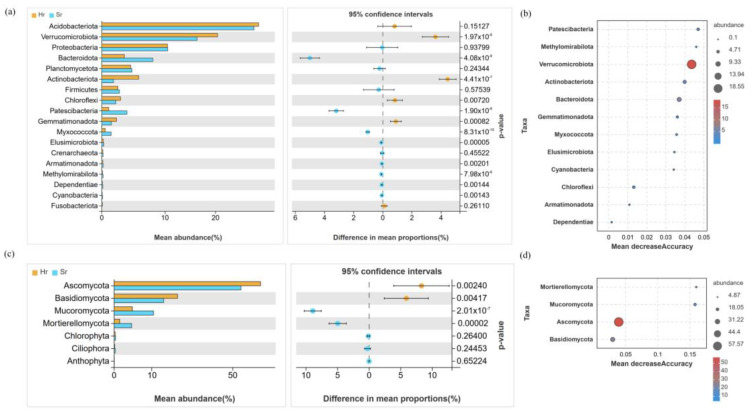
The relative abundance of bacterial (**a**) and fungal (**c**) communities at the phylum level under different respiratory patterns, as well as the random forest analysis results for bacteria (**b**) and fungi (**d**).

The bacterial community is predominantly composed of Acidobacteriota (26.81~27.64%), Verrucomicrobiota (16.78~20.38%), and Proteobacteria (11.58~11.63%). Notably, the relative abundance of Actinobacteria and Verrucomycota in the Hr group was significantly higher compared to that in the Sr group, whereas the relative abundance of Bacteroidota was significantly lower in the Hr group (*p* < 0.05) (Figure 4a). The fungal community primarily consists of Ascomycota (53.43~61.79%), Basidiomycota (20.90~26.73%), and Mucoromycota (7.64~16.64%) (Figure 4c). Following root removal, the Hr group exhibited a significant increase in the relative abundance of Ascomycota and Basidiomycota, while the relative abundance of Mucoromycota was significantly reduced (*p* < 0.05). The random forest analysis further confirmed the dominance of key functional microbial groups (Figure 4b,d). The richness analysis indicated that Verrucomycota and Ascomycota play crucial roles within the entire microbial community. Additionally, the heat map revealed complex interrelationships between these two taxa and other microorganisms.

**Figure 5:**
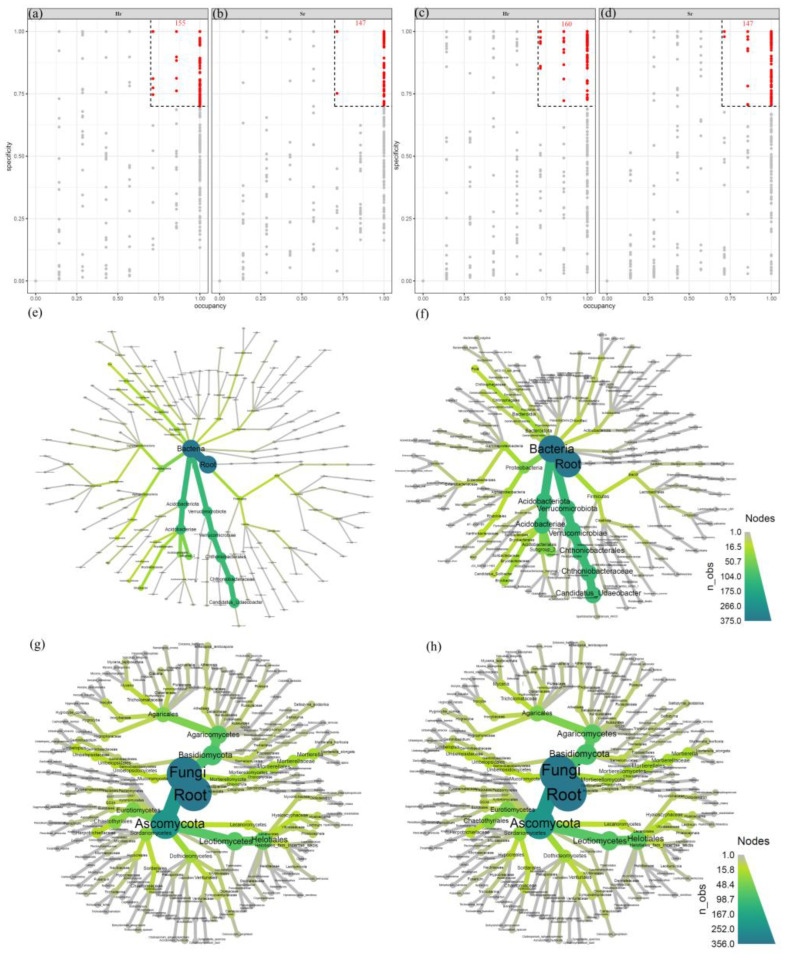
The microbial distribution associated with Sr and Hr: specificity-occupancy plots of bacteria (**a**) and fungi (**c**) involved in Hr; bacteria (**b**) and fungi (**d**) involved in Sr; taxonomic trees of bacteria (**e**) and fungi (**g**) contributing to Hr; and taxonomic trees of bacteria (**f**) and fungi (**h**) contributing to Sr.

The results of the occupancy and specificity analysis (Figure 5) indicate that 155 specialized bacterial species were identified in the Hr group, compared to 147 in the Sr group. The unique microbial populations in both groups predominantly belonged to the phyla Acidobacteriota and Verrucomicrobiota. With regard to the fungal community, the Hr group contained 160 specialized fungal species, whereas the Sr group contained 147. However, the core microbial communities of the two groups exhibited a high degree of similarity. These findings suggest that the bacterial community demonstrates specificity, while the fungal community appears to be more homogenized. The observed conservation of the fungal community supports its essential role in the process of carbon stabilization.

**Figure 6:**
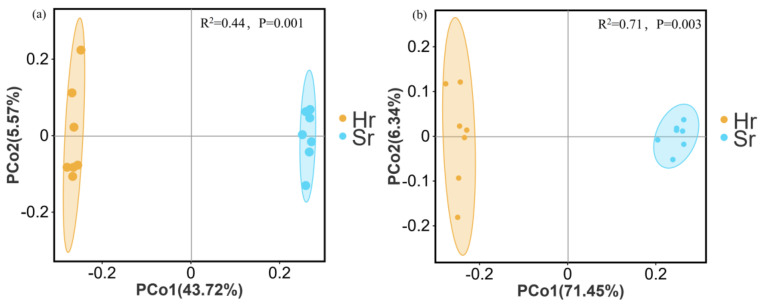
PCoA analysis of β-diversity in microbial communities: bacterial (**a**) and fungal communities (**b**).

PCoA based on Bray–Curtis distance demonstrates that the microbial communities in Hr and Sr plots exhibit significant spatial separation. As shown in Figure 6a, the bacterial community’s first principal component accounts for 43.72% of the variance, while the second axis explains 5.57%. Similarly, as illustrated in Figure 6b, the first principal component of the fungal community explains 71.45% of the variation, with the second axis accounting for 6.34%. Results from the multivariate permutation analysis of variance (PERMANOVA) indicate that root removal significantly influences the community structures of both bacteria (R^2^ = 0.44, *p* = 0.001) and fungi (R^2^ = 0.71, *p* = 0.003).

**Figure 7:**
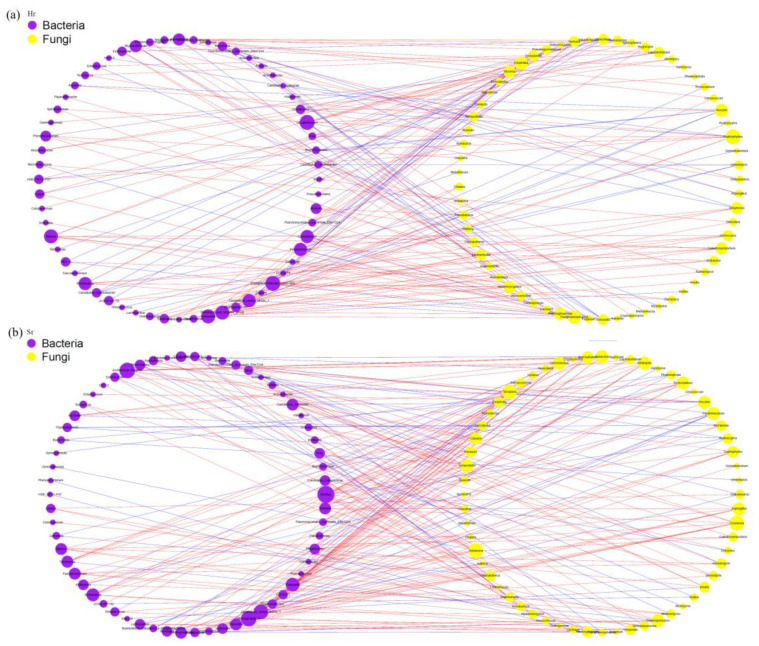
Network analysis between bacteria and fungi: (**a**) Hr; (**b**) Sr. (The red lines indicate positive correlations between ASVs, whereas the blue lines denote negative correlations.)

Based on the Spearman rank correlation coefficient (r > 0.6, FDR-corrected *p* < 0.01), ASVs with an occurrence rate of ≥80% were selected to construct a co-occurrence network. Topological visualization was conducted using Cytoscape v3.8.0 (Figure 7a,b), where nodes represent ASV-based operational taxonomic units and edge weights reflect the strength of association between species. The network analysis reveals that both Hr and Sr systems exhibit a predominantly positive co-occurrence pattern, with positively correlated edges accounting for 70.37% and 71.36%, respectively. Furthermore, the co-occurrence network in Sr soil demonstrates a higher average degree, a greater number of key species, fewer modular structures, and an increased number of co-occurring associations.

**Figure 8:**
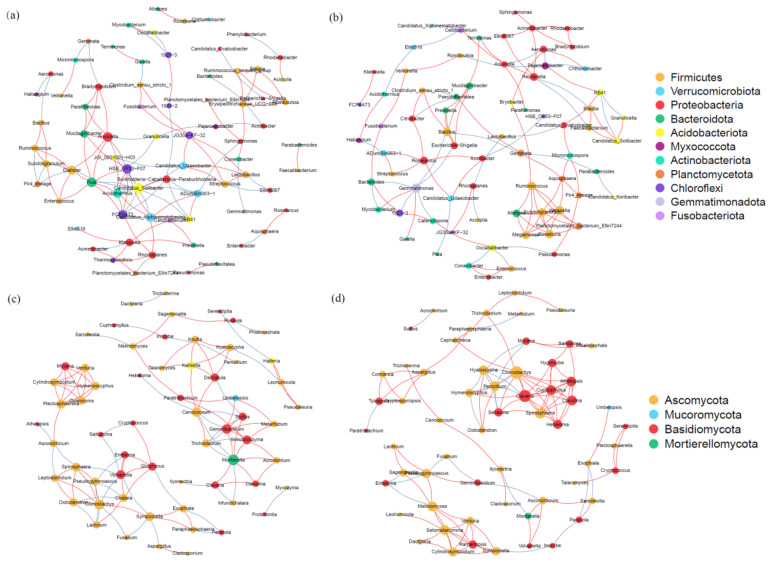
Co-occurrence patterns of microbial associations between bacterial communities (**a**) and fungal communities (**c**) in Hr, and between bacterial communities (**b**) and fungal communities (**d**) in Sr. (The red lines indicate positive correlations, whereas the blue lines denote negative correlations.)

The soil microbial network (Figure 8) consists of 266 nodes (ASVs) and 436 edges. The average path length between all node pairs was calculated as 4.34 edges, with a maximum distance (diameter) of 14 edges, suggesting the presence of multi-level interaction patterns among microorganisms. The network exhibits an aggregation coefficient of 0.48 and a modularity index of 0.69, indicating that it possesses significant modular structural characteristics. Furthermore, positive co-occurrence relationships are more prevalent than negative ones, accounting for over 60% of all interactions. A comparative analysis of the Hr and Sr systems reveals notable differences. Specifically, the bacterial and fungal networks within the Hr system exhibit a significantly higher number of edges and a shorter average path length compared to those in the Sr system, suggesting stronger microbial interactions and enhanced efficiency of information transfer in the Hr system.

### 3.4. Microbial Community Assembly Process

**Figure 9:**
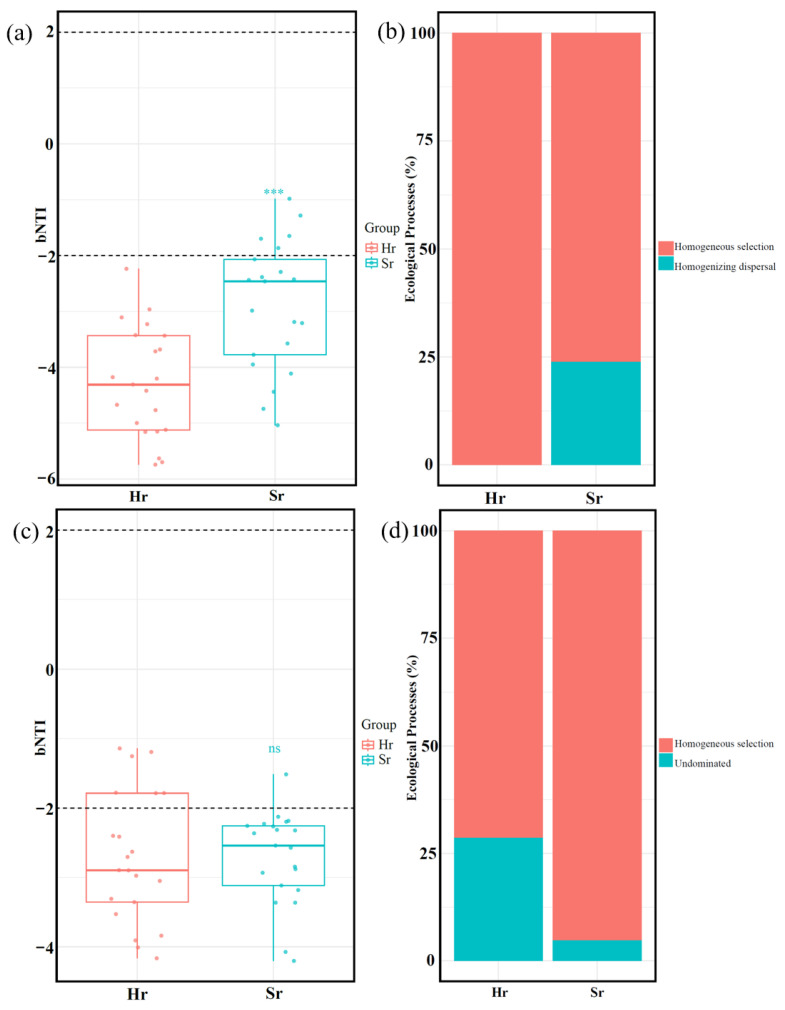
The assembly processes of microbial communities. Distribution of βNTI values in bacterial (**a**) and fungal (**c**) communities; contribution of various ecological processes to the assembly of bacterial (**b**) and fungal (**d**) communities. *** *p* < 0.001, ns: No significant difference.

The ecological processes of microbial communities with distinct respiratory patterns were analyzed using the null model. βNTI results indicated that deterministic processes predominantly governed community assembly in both Hr and Sr systems. Notably, the intensity of deterministic effects was significantly higher in Sr bacterial communities compared to Hr bacterial communities (*p* < 0.05). However, fungal deterministic effects in Hr and Sr systems were not statistically significant (Figure 9a,c). RCbray analysis (Figure 9b,d) revealed that homogeneous selection contributed more strongly to the bacterial community in Hr (100%) than in Sr (76.19%). Conversely, the fungal community exhibited an opposite trend, with Hr contributing 71.43% and Sr contributing 95.24% (*p* < 0.01). Furthermore, the effect sizes of homogenizing dispersal in the bacterial community and undominated processes in the fungal community were markedly lower than those of homogeneous selection. In particular, in the Sr fungal community, the contribution rate of undominated processes was as low as 4.76%. These findings suggest that community formation is primarily driven by environmental filtering and species adaptability rather than stochastic processes such as random dispersal or ecological drift, indicating that the study area likely experiences relatively homogeneous abiotic conditions.

### 3.5. Contribution of Environmental Variables to the Structure of Microbial Communities

**Figure 10:**
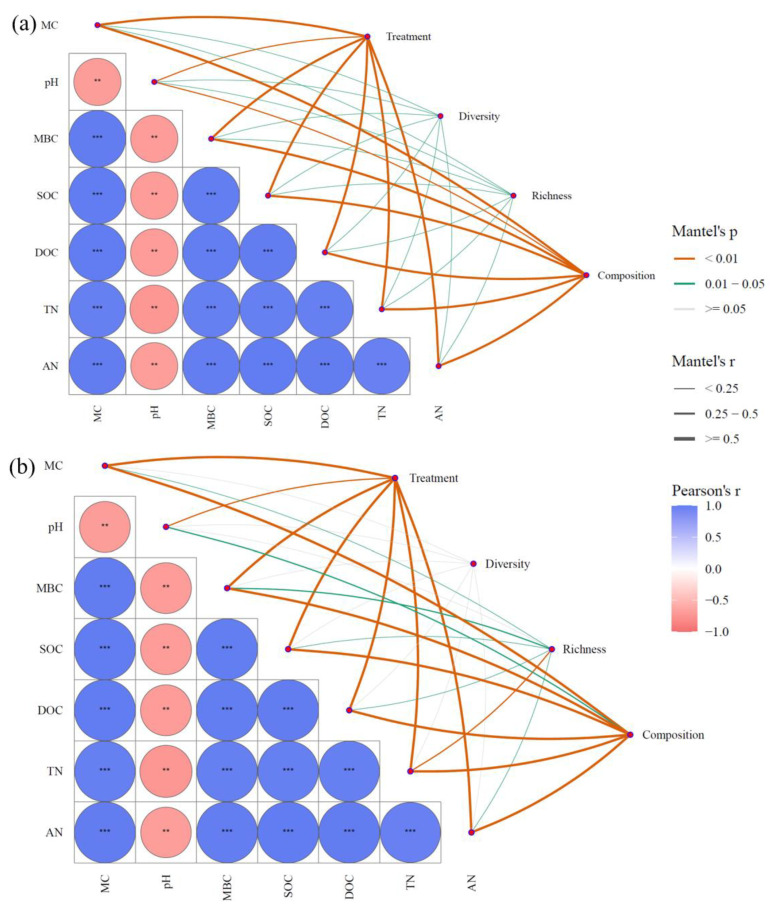
Mantel test for environmental factors and microorganisms: the relationship between bacteria (**a**) and fungi (**b**) and environmental factors. (** *p* < 0.01, *** *p* < 0.001).

Based on the Mantel test analysis, root removal treatment was found to significantly influence environmental variables. Furthermore, the regulatory effect of environmental variables on the composition, diversity, and species richness of bacterial communities was significantly greater than that observed for fungal communities (*p* < 0.05; Figure 10). MC, pH, MBC, SOC, DOC, AN, and TN were all significantly associated with the structure of both bacterial and fungal communities (*p* < 0.05). Notably, pH exhibited a particularly strong regulatory influence and showed significant negative correlations with MC, MBC, SOC, DOC, AN, and TN. This suggests that pH levels may substantially alter other environmental factors, thereby indirectly influencing microbial community composition.

## 4. Discussion

### 4.1. Regulation of Environmental Factors by Microbial Functional Groups

Soil microorganisms are crucial in forest ecosystems for nutrient cycling, soil quality enhancement, tree growth support, and ecosystem stability. Key physicochemical properties like pH and C/N ratio drive microbial community composition and activity [26]. The Verrucomicrobiota is widely distributed in soil environments and plays a significant ecological role [27]. This research indicates that the relative abundance of Verrucomicrobiota increases markedly during Hr, which may be attributed to its strong capacity for degrading macromolecular organic substances [28]. The substantial presence of organic matter in soil not only serves as a primary metabolic substrate supporting the carbon cycle for microorganisms such as Verrucomicrobiota, but members of this phylum can also regulate the quality and quantity of organic matter, thereby shaping the habitat conditions and limiting the proliferation of eutrophic bacteria [29]. Following the removal of the root system, autotrophic respiration—comprising root metabolism and rhizosphere microbial activity—is effectively eliminated. Concurrently, members of the Verrucomicrobiota within the heterotrophic microbial community become the dominant group due to their high efficiency in decomposing recalcitrant carbon compounds, such as lignin derivatives and plant residues. This shift leads to a significant reduction in SOC. Furthermore, given their oligotrophic nature, these microorganisms contribute to a decrease in DOC accumulation [30,31]. The Actinobacteria represent one of the primary microbial groups involved in the decomposition of cellulose and lignin in soil environments. This group is capable of surviving under relatively nutrient-poor conditions. However, its competitive advantage diminishes in root zones characterized by high organic carbon availability. Under conditions of organic carbon limitation, the relative abundance of Actinobacteria tends to increase [32]. Furthermore, certain bacterial taxa within the Verrucomicrobiota and Actinobacteria are known to produce alkaline compounds such as ammonia through ammonification processes, which can contribute to an elevation in soil pH [33].

Fungal communities demonstrate greater metabolic advantages. The relative abundances of Ascomycota and Basidiomycota involved in Hr are higher compared to Sr in this research. Compared to bacteria, fungi (especially Ascomycota and Basidiomycota) exhibit notable advantages in the decomposition efficiency of recalcitrant carbon compounds (lignin, keratin, cellulose, etc.) [34,35]. As a representative group of saprophytic fungi, they primarily secrete extracellular enzymes (e.g., oxidases, hydrolases) to break down complex organics. Ascomycota play a crucial role in the mineralization of complex organic materials, such as keratin and lignin [36]. At the same time, in addition to forming symbiotic relationships with plants, members of the Basidiomycota phylum secrete multiple hydrolytic enzyme systems that can effectively facilitate the decomposition and transformation of complex organic compounds such as cellulose [37]. In environments dominated by heterotrophic respiration, these two fungi typically exhibit higher abundance. The synergistic interaction between the two phyla significantly accelerated the hydrolysis of structural polysaccharides in plant litter and ultimately contributed to a decline in organic matter stocks within the ecosystem by suppressing the formation of inert carbon pools [38,39]. It can be inferred from this observation that the dynamic abundance of microbial communities influences the evolution of soil environmental factors by modulating the degradation processes of recalcitrant carbon and the intensity of ammonification.

### 4.2. Transformation of Microbial Metabolic Strategies Under Heterotrophic Respiration

The root system introduces labile carbon into the soil through photosynthetic carbon allocation, such as root exudates and rhizosphere deposits. This process stimulates the rapid proliferation of rhizosphere microorganisms, forming a “microbial pump” that enhances the accumulation of SOC. Concurrently, root uptake of nitrogen (AN and TN) reduces microbial N competition, sustaining elevated levels of MBC and AN [40,41]. However, upon removal of roots, microorganisms shift their activity towards the decomposition of SOC. At this stage, eutrophic r-strategist taxa (e.g., Proteobacteria and Bacteroidota) exhibit reduced abundance due to the depletion of carbon sources previously supplied by root exudates [42]. In contrast, oligotrophic K-strategist taxa (e.g., Verrucomicrobiota) demonstrate notable adaptability in nutrient-limited environments following root removal [43]. These fungi maintain efficient energy acquisition at low metabolic rates by upregulating genes for recalcitrant carbon-degrading enzymes (e.g., cellulase, xylanase). This shift in metabolic strategy was similarly pronounced in fungal communities, where the relative abundance of Ascomycota and Basidiomycota increased significantly following the root removal. This type of fungi efficiently degrades lignin–cellulose complexes through the secretion of multi-enzyme synergistic systems, including lignin peroxidase (LiP), manganese peroxidase (MnP), and cellulases such as cellobiohydrolase (CBH) and endoglucanase (EG) [44,45]. Compared with R-strategy microorganisms that depend on readily decomposable carbon sources, this fungus exhibits higher substrate affinity and lower maintenance metabolic requirements, thereby maintaining a competitive advantage in soil environments where DOC is limited. Variations in the soil’s physicochemical properties correspond well with the results obtained in this study. The transformation in the structure and function of the aforementioned microbial community suggests that the soil system has transitioned from a “root-driven carbon input and stabilization system” to a “net carbon loss system dominated by recalcitrant carbon”.

### 4.3. Response Mechanisms of Microbial Community Assembly to Environmental Factors

Research indicates that the community assembly mechanisms often exhibit scale dependence. At smaller spatial scales, physical barriers to microbial dispersal are relatively minimal, making environmental selection and homogeneous dispersal the predominant processes. In contrast, at larger spatial scales, dispersal limitation and ecological drift tend to play more significant roles [46]. Intense homogeneous selection in this study indicates strong, uniform environmental filtering, where similar environmental filters and convergent resource availability shape both bacterial and fungal communities. Key drivers include the dominance of soil physicochemical properties, convergence in microbial interaction networks, and minimal influence of spatial scale and dispersal limitations, collectively resulting in stringent niche-based selection; only well-adapted taxa were able to persist [47,48,49]. Meanwhile, in nutrient-limited environments, microorganisms establish dominant ecological niches through the optimization of metabolic strategies, leading to competitive exclusion effects [50,51]. Certain functional groups (e.g., oligotrophic Verrucomicrobiota) can mitigate the influence of stochastic processes on community assembly by secreting inhibitory metabolites or preferentially utilizing essential resources. Such competitive pressures significantly diminish the random component in community structuring. Overall, the predominance of homogeneous selection indicates the central role of environmental homogenization and functional trait filtering, whereas the relatively low effect size of homogeneous dispersal and other non-dominant processes may be attributed to spatial scale constraints and functional redundancy.

While this study provides insights into how the vegetation clearance impacts microbial communities of soils in cold-temperate forests, several limitations should be noted:

(1) Seasonal sampling bias may affect microorganism abundance patterns, warranting multi-temporal validation;
(2) Functional inferences lack metagenomic validation; future studies should integrate multi-omics approaches to address these limitations.

## 5. Conclusions

This study conducted field experiments within the Huzhong National Nature Reserve. By analyzing variations in soil physicochemical properties, including MC, pH, MBC, SOC, DOC, TN, and AN, as well as microbial biomass and community structure at sites exhibiting Sr and Hr, this research elucidated the distinct regulatory mechanisms through which soil respiration differentiation influences microbial community assembly. (1) Following the removal of vegetation, the physical and chemical properties of the soil exhibit a heterogeneous pattern. The biomass of microorganisms that predominantly contribute to Hr declines, accompanied by changes in the relative abundance of specific microbial taxa, including phyla such as Verrucomicrobiota, Ascomycota, and Basidiomycota. (2) The assembly pattern of microbial communities is primarily governed by homogeneous selection, with additional contributions from homogeneous dispersal and stochastic processes. (3) The microbial interaction network within the Hr exhibits high complexity and short path lengths, indicating a greater degree of functional redundancy and resilience in its carbon turnover process. (4) Environmental factors promote community differentiation through a dual mechanism: directly influencing microbial community structure and indirectly modifying soil chemical properties. This drives community differentiation and corresponding differences in respiratory function. This study presents a theoretical framework for understanding the microbial regulatory mechanisms underlying soil carbon sink function in cold-temperate forests and provides a foundation for the precise prediction of soil respiration differentiation effects.

## Figures and Tables

**Table 1:** Physical and chemical properties of soil.

Sample	MC %	pH	MBC mg/kg	SOC g/kg	DOC mg/kg	TN g/kg	AN mg/kg
Sr	18.89 ± 0.12 ^a^	3.97 ± 0.11 ^b^	880.40 ± 15.70 ^a^	47.46 ± 0.34 ^a^	74.23 ± 1.17 ^a^	3.18 ± 0.06 ^a^	253.80 ± 2.81 ^a^
Hr	14.28 ± 0.09 ^b^	4.64 ± 0.48 ^a^	549.07 ± 17.12 ^b^	27.46 ± 0.3 ^b^	41.30 ± 0.69 ^b^	2.25 ± 0.11 ^b^	142.88 ± 3.43 ^b^
*p*-value	*p* < 0.001	*p* = 0.006	*p* < 0.001	*p* < 0.001	*p* < 0.001	*p* < 0.001	*p* < 0.001

Different letters represent significant differences (*p* < 0.05); the same below.

## Data Availability

The data have been deposited in the National Center for Biotechnology Information Sequence Read Archive under accession number PRJNA1290219.
